# Increasing the information rates of optical communications via coded modulation: a study of transceiver performance

**DOI:** 10.1038/srep21278

**Published:** 2016-02-11

**Authors:** Robert Maher, Alex Alvarado, Domaniç Lavery, Polina Bayvel

**Affiliations:** 1 Optical Networks Group, Department of Electronic & Electrical Engineering, UCL (University College London), London WC1E 7JE, UK

## Abstract

Optical fibre underpins the global communications infrastructure and has experienced an astonishing evolution over the past four decades, with current commercial systems transmitting data rates in excess of 10 Tb/s over a single fibre core. The continuation of this dramatic growth in throughput has become constrained due to a power dependent nonlinear distortion arising from a phenomenon known as the Kerr effect. The mitigation of fibre nonlinearities is an area of intense research. However, even in the absence of nonlinear distortion, the practical limit on the transmission throughput of a single fibre core is dominated by the finite signal-to-noise ratio (SNR) afforded by current state-of-the-art coherent optical transceivers. Therefore, the key to maximising the number of information bits that can be reliably transmitted over a fibre channel hinges on the simultaneous optimisation of the modulation format and code rate, based on the SNR achieved at the receiver. In this work, we use an information theoretic approach based on the mutual information and the generalised mutual information to characterise a state-of-the-art dual polarisation *m*-ary quadrature amplitude modulation transceiver and subsequently apply this methodology to a 15-carrier super-channel to achieve the highest throughput (1.125 Tb/s) ever recorded using a single coherent receiver.

Optical communications technology has demonstrated unprecedented development over the past three decades and now stands alone as the enabling technology that underpins the global information infrastructure. Over this period, the throughput (measured in bits per second) of lightwave communications systems increased from 100 Mb/s in 1970 to 10 Tb/s in present day commercial systems, which represents an astonishing 100,000-fold increase. The key technologies that fuelled this surge in capacity were wavelength division multiplexing (WDM), improved fibre types, optical amplification and coherent optical networking. Although the introduction of Erbium and Raman fibre amplifiers negated the need for electronic regenerators and enabled dense WDM transmission, the success and performance of these amplifier technologies has effectively constrained the useable fibre bandwidth (BW) to approximately 10–15 THz, which is now beginning to limit the maximum throughput of optical systems. Within this BW, the throughput can only be increased by reducing the spectral guard bands between WDM carriers, increasing the information rate (IR, measured in bits per symbol) of the modulation format or through new forward error correction (FEC) schemes that require lower redundancy without loss in performance. The combination of these techniques aim to increase the spectral efficiency (SE) of each WDM carrier. However, for Nyquist spaced WDM systems, increasing the SE is highly dependent on the IR, which inherently scales with received signal-to-noise ratio (SNR). Currently, the upper limit on the available SNR in a coherent optical transmission system, in the absence of fibre nonlinearity, is bounded by the transceiver subsystems.

Coded Modulation (CM), which is the combination of multi-level modulation and FEC, is often coupled with spectral shaping to constrain the bandwidth of optical carriers and is a fundamental methodology applied to maximise the IR of a SNR-limited optical communications system. Figure 1(a) illustrates an example of a generic CM system. The CM transmitter (CM TX) can be seen as a non-binary encoder that maps a sequence of uniformly distributed information bits 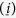 of length 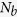 to sequences of vectors 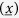 of length 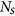 (The information bits are assumed to have already been passed through a source encoder for data compression.). Each vector of complex symbols belongs to the codebook, 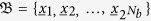, where 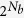 is the number of possible transmitted messages. The (uniformly distributed) sequence of symbols is subsequently transmitted over the optical channel, which includes a silica core optical fibre and the transceiver subsystems, which includes the corresponding digital signal processing (DSP) implementation. The CM receiver (CM RX) attempts to estimate the transmitted vector, 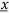, from the received noisy symbols 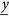.

Information theory addresses fundamental questions regarding the maximum amount of information that can be reliably transmitted through any channel^1^,^2^. The mutual information (MI) between the transmitted and received symbols is a key quantity in information theory, as it represents the largest achievable IR (A rate is said to be achievable if there exist an encoder operating at that rate and a decoder giving a vanishing error probability as the block length tends to infinity.) for a CM system based on the optimal maximum likelihood (ML) sequence decoder. The MI has been analysed in the context of the optical channel in e.g.^3^,^4^, and has been previously employed as a performance metric for digital optical communications systems^5^,^6^. Recently, experimental demonstrations have verified the use of MI to predict the post soft decision FEC (SD-FEC) bit error ratio (BER_post_) in optical systems based on the dual polarisation (DP) quadrature phase shift keyed (QPSK) format^7^, while the MI has also been used as a figure of merit for WDM transmission systems based on the *m*-ary quadrature amplitude modulation (QAM) format^8^,^9^.

Typically, the CM TX in Fig. 1(a) is implemented as a concatenation of a binary encoder and a memoryless mapper. In this CM TX architecture, shown in Fig. 1(b), information bits are passed into the binary encoder, which adds redundancy (for error correction at the receiver) and operates at a code rate, 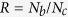, where 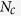
is the number of coded bits 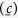. The coded bits are mapped to a set of discrete constellation points using a memoryless mapper and are subsequently transmitted over the optical channel. Although the ML sequence decoder can be used for this transmitter architecture, the high complexity associated with this decoder currently renders its use impractical for commercial systems. Therefore, a more feasible (albeit sup-optimal) receiver is to use a simple two-step decoding process that decouples the decoder and demapper into two distinct processes. This bit-wise receiver structure is the key functional block in a bit-interleaved coded modulation (BICM) based system^10^, which is illustrated in Fig. 1(b). The BICM receiver (BICM RX) consists of a memoryless demapper and a SD-FEC decoder. The demapper calculates ratios of bit probabilities (known as log likelihood ratios (LLRs)), 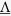, that are passed into the binary decoder and are used to estimate the transmitted bits. The decoded bits are subsequently used to calculate the post-SD-FEC BER, while the pre-SD-FEC BER (BER_pre_) is calculated by passing the received symbols through a hard decision (HD) demapper. These two BERs are schematically shown in Fig. 1(b). As the BICM receiver structure operates on bits rather than symbols, the MI is not an achievable IR for this CM architecture^11^. Conversely, the generalised mutual information (GMI), which is calculated using the coded bits and the LLRs, is an achievable IR for a BICM system and thus, provides a more accurate prediction of the performance of commercial optical systems that employ bit-wise decoders. The GMI has previously been demonstrated as a figure of merit in the optical communications domain, both through numerical simulations^11^,^12^ and experimental demonstrations^13^, while it has also been proposed as an accurate indicator of the BER_post_^14^.

The main contribution of this work is to use information theoretic quantities to demonstrate that the upper limit on the throughput of any optical transmission system is ultimately limited by the finite signal-to-noise ratio (SNR) of the digital coherent transceiver subsystems. In particular, we show that the modulation format and code rate of a practical BICM optical system must be simultaneously optimised to match the specific SNR achieved after digital coherent detection. This experimental characterisation shows that the industry standard DP-QPSK modulation format should only be employed in a transmission system if the received SNR is <2.5 dB. Furthermore, a comparison of single rate and variable rate low density parity check (LDPC) SD-FEC schemes is presented for a 15 sub-carrier super-channel system. It is shown that the variable rate LPDC scheme provides a 15.3% increase in throughput, thus achieving a net bit rate of 1.125 Tb/s, which is the largest throughput ever recorded using a single receiver.

## Results

### Coherent Optical Transceiver Performance Analysis

The single wavelength back-to-back (B2B) DP-*m*QAM optical transceiver experimental test-bed used in this work is shown in Fig. 2(a). The in-phase (I) and quadrature (Q) drive signals required for each *m*QAM format, where 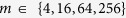, were generated offline (as detailed in the Methods section) before being loaded onto a pair of field programmable gate arrays (FPGAs) and output using two digital-to-analogue convertors (DACs) operating at 32 GSa/s (4 Sa/sym). Each electrical signal was amplified using a linear electrical amplifier and passed through an 8^th^-order Bessel low pass filter (LPF) with a rejection ratio in excess of 20 dB/GHz and a 3 dB bandwidth of 6.2 GHz, before being applied to an IQ modulator. The output of an external cavity laser (ECL) with a 1.1 kHz linewidth was passed directly into the modulator before being optically amplified and polarisation multiplexed to form an 8 GBd Nyquist shaped DP-*m*QAM optical carrier. The DP-*m*QAM signal was passed directly into the signal port of the digital coherent receiver, which had a sample rate of 160 GSa/s and an analogue electrical bandwidth of 62.5 GHz. Amplified spontaneous emission (ASE) noise was added to the signal to vary the received SNR and a second ECL (1.5 kHz linewidth) was used as a local oscillator (LO). The key blocks of the blind DSP implementation, including the SNR, MI and GMI estimation, are illustrated in Fig. 2(b) and are described in detail in the Methods section. The LDPC decoder (followed by a 6.25% overhead (OH) HD- FEC decoder) was implemented offline to calculate the BER_post_ and was identical to that described in the Methods section of^15^.

The MI was calculated over both polarisations as a function of the estimated SNR for 4, 16, 64 and 256QAM respectively and is shown in Fig. 3(a). The experimental measurement was recorded by adding ASE noise to the signal and measuring the MI for 50 discrete values of SNR ranging from 0 dB to 24 dB. The capacity of an additive white Gaussian noise (AWGN) channel, 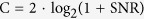, is also shown to provide a performance reference relative to the experimental results (The factor of 2 before the log accounts for the use of two polarisations.). For DP-4QAM (QPSK), the MI increased from 1.95 b/sym at a received SNR of 0 dB, to 3.17 b/sym at a SNR of 4 dB and finally to a maximum MI of 4 b/sym (2 b/sym for 4QAM over two polarisations) at a SNR of 12 dB. Similar performance was achieved for the DP-16QAM format, which exhibited a MI of 1.98 b/sym at a SNR of 0 dB and achieved a maximum of 8 b/sym at a SNR of 19.5 dB. The DP-64QAM format realised the maximum MI of 12 b/sym at a SNR of 24 dB, however it is evident from Fig. 3(a) that the DP-256QAM format never achieved the maximum possible MI of 16 b/sym.

The MI for the DP-256QAM format was limited to 14.7 b/sym because of a saturation in the SNR within the coherent optical transceiver. Therefore, as the power of the ASE noise loading stage was reduced towards zero, the SNR remained constant at 24 dB. The main contributions to the limited SNR within the digital coherent transceiver were the DAC and linear electrical amplifiers in the transmitter and the analogue-to-digital convertors (ADC) in the real time sampling oscilloscope at the receiver. The DAC used in this work was a Micram Vega DAC 2, which exhibited a physical resolution of 6 bits. However, the measured effective number of bits (ENOB) of the DAC was ~5 bits over the frequency range from 0 to 4 GHz (bandwidth of the spectrally shaped IQ drive signals), which corresponds to a SNR of ~32 dB. The linear amplifiers had a noise figure of 6 dB at a frequency of 4 GHz, therefore the maximum attainable SNR from the electrical components within the transmitter was ~26 dB. The ADCs in the real time sampling oscilloscope also exhibited a frequency dependent ENOB, which was 4.8 bits at a frequency of 4 GHz (reduced to 4.3 bits at a frequency of 60 GHz). The additional loss in SNR was due to the inclusion of the optical components and a non-ideal blind DSP implementation.

It is important to note that the MI curves shown in Fig. 3(a) never intersect. Therefore, in a transceiver limited CM optical communications system, the largest constellation size will always provide the greatest achievable IR and therefore the highest throughput. However, the MI is not an achievable IR for BICM optical systems, therefore we must consider the GMI. The corresponding experimentally measured GMI as a function of received SNR for all four square QAM modulation formats is shown in Fig. 3(b). The DP-4QAM format achieved identical performance to the MI characterisation. This is because Gray-labelled DP-4QAM can be decomposed into two independent binary phase shift keyed formats, which is the only case where the GMI is equal to the MI^11^,^14^. In the medium-to-high SNR regimes of the DP-16QAM, DP-64QAM and DP-256QAM formats, negligible penalty is observed between the GMI and MI, achieving a GMI of 8 b/sym, 12 b/sym and 14.7 b/sym at received SNRs of 19.53 dB, 24 dB and 24 dB, respectively. However, a small penalty was incurred for each format (with the exception of DP-4QAM) in the low SNR regime relative to the MI, which is attributed to the sub-optimality of BICM decoders. Significantly, it is also evident from this characterisation that the GMI curves intersect, thus providing specific SNR regimes where one particular modulation format exhibits the greatest achievable IR. Therefore, the key to maximising the throughput of a transceiver limited BICM system hinges on the correct choice of modulation format and code rate, which is ultimately dependent on the received SNR of each optical carrier after digital coherent detection.

Although the GMI provides the optimum modulation format as a function of received SNR, it does not directly relate to the code rate 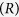 of the binary encoder. However, the normalised GMI, obtained by dividing the recorded GMI for each format by 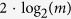, provides an upper bound on the code rate (lower bound on the FEC overhead) that can be used to reliably transmit information through the channel for a given SNR. Figure 4 shows the maximum code rate as a function of the received SNR for all four formats (The GMI has not been proven to be the largest achievable IR for BICM. However, the GMI is known to predict well the performance of CM transceivers based on capacity-approaching SD-FEC decoders.). The dashed lines illustrate the optimum SNR when the constellation size should be increased in order to ensure that the IR increases monotonically with SNR. In the low SNR regime (<2.5 dB) it is more efficient to use the DP-4QAM format, however as the SNR is increased to 2.5 dB, it becomes more spectrally efficient to use the DP-16QAM format with a code rate of 0.34 (corresponding to a FEC OH of 194.12%), rather than the DP-4QAM format with rate 0.679 (FEC OH: 47.27%). This is a key observation and demonstrates that depending on the SNR performance of a given BICM system, the net bit rate can be maximised by considering very high FEC OHs, which far exceed that of currently installed optical communications systems that typically reserve an overhead ranging from 7% to ~20% for channel coding^16^. The experimental characterisation shown in Fig. 4 also demonstrates that the industry standard modulation format for long haul communications, DP-4QAM, should only be used if the received SNR is <2.5 dB. The optimal modulation format switches to DP-64QAM at a SNR of 9.8 dB and a code rate of 0.52 (FEC OH: 92.31%) and finally switches to the DP-256QAM format for any received SNR greater than 16.63 dB.

The characterisation presented in this section can be applied to any transceiver subsystem and provides an accurate indication of the maximum possible performance in terms of throughput. This optimisation of the modulation format and code rate as a function of SNR is especially pertinent for future terabit transmission systems that employ optical super-channels that contain sub-carriers with a non-uniform SNR profile.

#### Tb/s Super-Channel Optical System

An optical super-channel consists of grouping a number of lower bandwidth sub-carriers that are collectively routed from transmitter to receiver and is a technique that has been proposed for next generation 400 Gb/s or 1 Tb/s transmission systems. Each of the sub-carriers that comprise the super-channel are independently generated at the transmitter and are typically detected using independent digital coherent receivers^17^. However, the use of high bandwidth coherent receivers for the simultaneous reception of an entire super-channel has been recently proposed and demonstrated experimentally^8^,^9^,^18^. Joint detection of a super- channel is beneficial as it reduces the number of required receivers and provides the possibility for joint signal processing, such as multi-channel digital back propagation^15^, joint carrier phase estimation or mitigation of linear crosstalk^19^. The record throughput achieved to date using a single coherent receiver is exactly 1 Tb/s and was recently demonstrated using a spectrally sliced transmitter and a digital coherent receiver with an optical bandwidth of 125 GHz (analogue electrical BW of 62.5 GHz)^20^. However, as previously mentioned, in order to ensure that the net throughput of a super-channel system is maximised, the code rate and modulation format must be simultaneously optimised. Therefore, the GMI performance characterisation demonstrated in the previous section was applied to a B2B 15 sub-carrier super-channel system to illustrate the relative gains in throughput afforded by this methodology.

The transmitter setup used for the super-channel characterisation is illustrated in Fig. 5. The output of the 1.1 kHz ECL was passed through an optical comb generator (OCG) that consisted of two cascaded Mach-Zehnder modulators, both overdriven with an amplified sinusoidal wave with a frequency of 8.1 GHz. This generated fifteen, evenly spaced, frequency locked comb lines, with the channel spacing equal to the frequency of the applied sine wave. A liquid crystal on silicon (LCOS) optical filter was used to achieve a comb flatness with a power variation of <1.5 dB and also to suppress the out-of-band sub-carriers. Figure 6(a) shows the 15 generated sub-carriers, at the output of the OCG stage. The out-of-band sub-carriers only achieved a suppression ratio of 5 dB due to the limited resolution of the LCOS filter. The total bandwidth of the super-channel was 121.5 GHz, which was within the maximum optical bandwidth of our digital coherent receiver (125 GHz). The frequency comb was subsequently separated into odd and even carriers using three cascaded Kylia micro-interferometer (MINT) interleavers. Each set of comb lines were independently modulated using two IQ modulators, with the corresponding spectra shown in Fig. 6(b,c). For the super-channel experiment, only the 256QAM format was considered and the required electrical in-phase and quadrature signals applied to the IQ modulators were generated identically to those detailed in the previous section. The modulated odd and even sub-carriers were decorrelated by 170 symbols before being combined and polarisation multiplexed to form a 15 sub-carrier 8 GBd DP-256QAM super-channel, with the corresponding spectrum shown in Fig. 6(d). The digital coherent receiver was identical to the setup described in the previous section and used the same high bandwidth photodiodes and real time sampling oscilloscope as in^20^. A single LO was used to receive all 15 sub-carriers simultaneously, with each sub-carrier individually down-converted to baseband in the digital domain (as shown in the dotted box in Fig. 2(b)) before the remaining DSP blocks (including SNR, MI and GMI estimation) and SD-FEC was performed. This ensured that the coherent receiver was operated as a true super-receiver, thus demonstrating the capability of the reception and demodulation of optical super-channels.

The experimentally measured B2B performance of the super-channel is shown in Fig. 7. A maximum MI of 12.4 b/sym was achieved for the central sub-carrier as shown in Fig. 7(a), which represents a degradation of 2.3 b/sym relative to the single channel performance outlined in the previous section. This penalty was due to a significant reduction in SNR of ~4 dB within the transmitter, which was caused by the introduction of the OCG stage and the comb flattening LCOS filter. This penalty could be avoided if free running lasers were employed within the transmitter. The MI performance of the super-channel degraded towards the outer sub-carriers, which was caused by a reduction in the received SNR due the intrinsic frequency dependent ENOB of the ADCs in the real time sampling oscilloscope. The worst performing sub-carriers were ±4, which experienced a significant dip in MI to 9.9 b/sym and 9.8 b/sym, respectively. The sharp deterioration in performance for these sub-carriers was again due to the intrinsic properties of the receiver ADCs. The sampling oscilloscope used in this work was an Agilent Infinium 62.5 GHz digital sampling oscilloscope (DSO). This DSO employed two interleaved 33 GHz ADCs to achieve the high BW 62.5 GHz module. Fine calibration and temperature stability was required to achieve low noise performance across the entire 62.5 GHz BW, however even minor variations in temperature resulted in a spike in the receiver noise at the interleaving frequency (33 GHz), which had a negative impact on the received SNR and thus MI of sub carriers ±4. The average MI across the entire super-channel was 10.94 b/sym, therefore indicating that a net bit rate of 1.312 Tb/s is possible using this transceiver setup.

The GMI performance was almost identical to the MI for the sub-carriers that exhibited the highest received SNR (−1, 0, 1, and 2). For the outer sub-carriers, the GMI incurred a penalty of ~0.25 b/sym. The mean GMI across the entire super-channel was 10.8 b/sym. This is a significant observation, as the MI provides the largest achievable IR for any coded modulation system, whereas the GMI provides an achievable IR for a practical and eminently realisable BICM system, however the difference in throughput between the MI and the GMI was only 1.3%, reducing from a possible bit rate of 1.312 Tb/s to 1.295 Tb/s. Therefore, this performance characterisation demonstrates that, if sufficient SNR is achieved at the receiver, a simple two-step BICM decoder will provide a level of performance that is only slightly degraded relative to the optimum ML receiver. The key to achieving the level of performance indicated by the GMI is determined by the continuous design and development of novel capacity achieving SD-FEC codes, which is an area of intense research. In order to demonstrate the performance penalty associated with using a standardised error correction code, we used a concatenated FEC scheme, as outlined in the Methods section of^15^.

The achieved IR after the FEC implementation was calculated using, 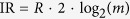, where 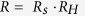
and 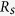 was the code rate for the inner LDPC code, while 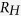 was the code rate for the outer (assumed) HD staircase code. The code rate, 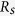, was independently optimised for each sub-carrier, based on the SNR at the receiver, in order to maximise the throughput. From Fig. 7(a), it is evident that a constant penalty in information rate of ~1.5 b/sym was incurred for all sub-carriers, thus providing a mean achieved information rate of 9.37 b/sym. The corresponding BER performance of each sub-carrier, before and after the SD-FEC decoder, is shown in Fig. 7(b). The BER_pre_ exhibits the characteristic performance that is dominated by the frequency dependent ADC resolution, with the central sub-carrier providing the lowest BER_pre_ (6.84·10^−2^) and sub-carriers ±4 incurring the highest BER_pre_ of 1.32·10^−1^ and 1.29·10^−1^, respectively. Adaptive code rates ranging from 0.54 to 0.72 were used across the super-channel, which resulted in a net bit rate of 1.125 Tb/s, which is the highest throughput ever reported for a single receiver and represents a significant increase of 12.5% relative to the previous record demonstrated in^20^.

## Discussion

The finite SNR of a coherent optical transceiver is an inherent property that cannot be mitigated or compensated and currently represents a significant obstacle to increasing the throughput of lightwave communications systems. The state-of-the-art transceiver used in this work provided a finite SNR of 24 dB, which enabled the generation and detection of the DP-256QAM format with a GMI of 14.7 b/sym. However, if practical improvements are made to the transceiver subsystems, greater information rates could be achieved. Figure 8 shows a simulation of the GMI as a function of the received SNR for dual polarisation square QAM formats from DP-4QAM up to DP-16384QAM. The dotted line indicates the saturated SNR (24 dB) of the transceiver used in this work and it is evident that the possible increase in throughput using higher order formats, such as DP-1024QAM or DP-4096QAM, is negligible relative to the performance achieved using the DP-256QAM format in this SNR-limited regime. However, if the SNR is unbounded, the achievable information rates continue to increase (at an approximate rate of 2 b/sym for every 3 dB increase in SNR) and the staircase characteristic demonstrated by the normalised GMI shown in Fig. 4 would extend to incorporate the higher order formats. Practically achieving a received SNR of up to or in excess of 50 dB is very challenging, however very high resolution DACs and ADCs that are capable of close to this level of performance are currently available, albeit at very low electrical bandwidths (~200 MHz).

A complementary approach to increase the information rate of a SNR limited transceiver involves the use of constellation shaping. Probabilistic and geometric shaping, which involve either using constellations with unequal per-symbol probabilities or constellations with unequally located points are two distinct concepts used to provide a sensitivity gain over conventional square QAM formats. A significant theoretical sensitivity enhancement of 1.53 dB is possible for asymptotically high orders of QAM, which would ensure that the digital coherent transceiver operates at an information rate close to capacity. Sensitivity gains of 0.8 dB (64QAM)^21^ and transmission reach enhancements of 43% (64QAM)^22^ have been very recently experimentally demonstrated for probabilistic shaping, while gains of up 1.3 dB (attributed to both constellation shaping and reduced quantisation noise)^23^ have been experimentally demonstrated for geometric shaping.

The importance of simultaneously optimising the modulation size and code rate was emphasised for the super-channel transmission system. For a non-adaptive FEC scheme, the code rate would have to be reduced sufficiently in order to adequately correct the bit errors for the worst performing sub-carrier, in order to achieve a BER below the the outer HD-FEC threshold. As shown in Fig. 7, the highest BER_pre_ of 1.32·10^−1^ was achieved for sub-carrier +4, which required a code rate of 0.54 for the inner LDPC decoder. If this code rate remained constant for all sub-carriers, the total throughout of the single receiver system would be 975 Gb/s. However, by optimising the code rate on a per-sub-carrier basis, the information throughput increased by 15.3% to 1.125 Tb/s, thus providing the highest ever throughput for a single coherent receiver. Ultimately, significant future increases in this record are reliant on the continual development of DACs, ADCs and implementable capacity-approaching FEC codes.

## Methods

### Digital Signal Processing

#### DSP at the Transmitter

**Figure 1:**
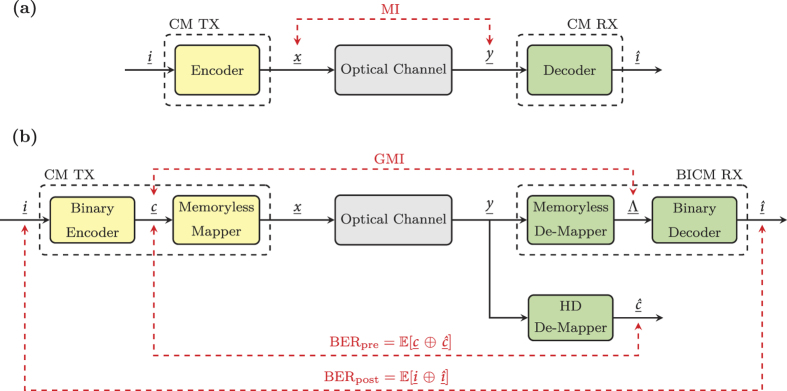
Coded modulation. (**a**) CM system using the optimal ML decoder. The MI is estimated using the transmitted and received symbols. (**b**) BICM system illustrating the variables used to measure the GMI, BER_pre_ and BER_post_.

**Figure 2:**
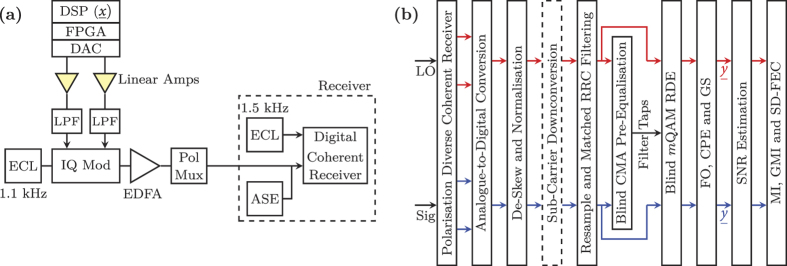
Single wavelength DP-*m*QAM transceiver. (**a**) Experimental setup and (**b**) DSP functions within digital coherent receiver.

**Figure 3:**
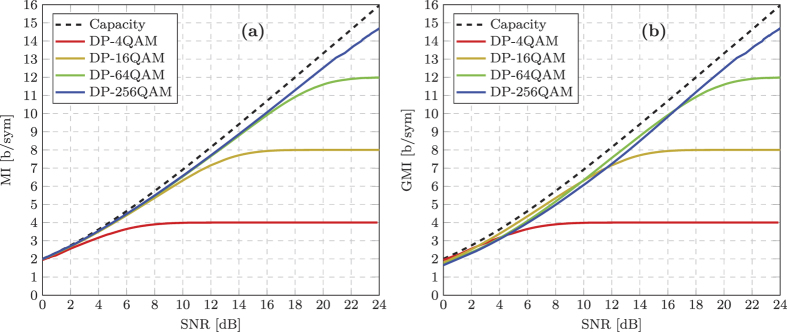
Experimentally measured MI and GMI as a function of received SNR. (**a**) MI (measured over two polarisations) for DP-4QAM (QPSK), DP-16QAM, DP-64QAM and DP-256QAM. The dotted line illustrates the capacity of an AWGN channel. (**b**) Corresponding GMI characterisation.

**Figure 4:**
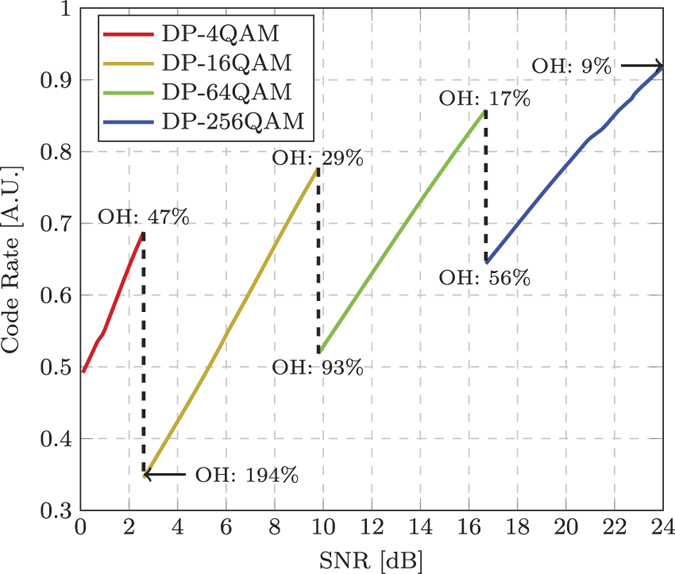
Experimentally measured normalised GMI, which provides the optimum modulation format and maximum code rate as a function of received SNR. The dotted lines indicate the optimum SNR to change the cardinality of the modulation format in order to achieve the maximum throughput. The corresponding FEC overheads at the transition points between modulation formats are also displayed.

**Figure 5:**
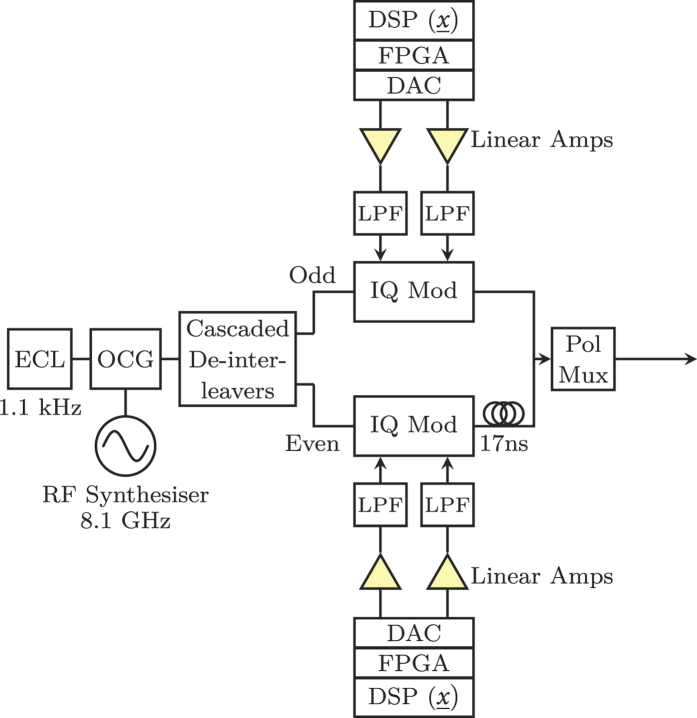
Experimental setup for the 15 sub-carrier super-channel transmitter.

**Figure 6:**
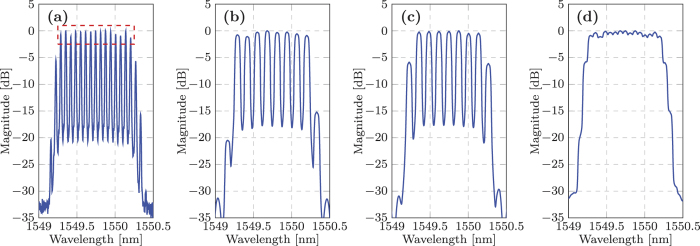
Optical spectra of the 15 sub-carrier super-channel. (**a**) Spectrum at the output of the OCG. The red dotted box illustrates the 15 sub-carriers used for this characterisation. (**b**) Spectrum of the modulated “Odd” carriers, at the output of the top IQ-modulator. (**c**) Spectrum of the modulated “Even” carriers, at the output of the bottom IQ-modulator. (**d**) Spectrum of the entire super-channel, at the output of the polarisation multiplexing stage.

**Figure 7:**
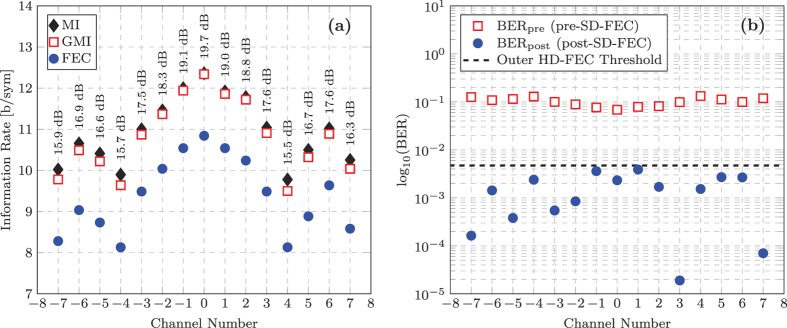
Experimentally measured performance of a 1 Tb/s DP-256QAM super-channel system. (**a**) Achievable information rate (as defined by MI and GMI) and the achieved information rate (obtained after the concatenated FEC scheme) as a function of channel number. The received SNR of each optical sub-carrier is also shown. (**b**) BER_pre_ and BER_post_, before and after the SD-FEC stage respectively.

**Figure 8:**
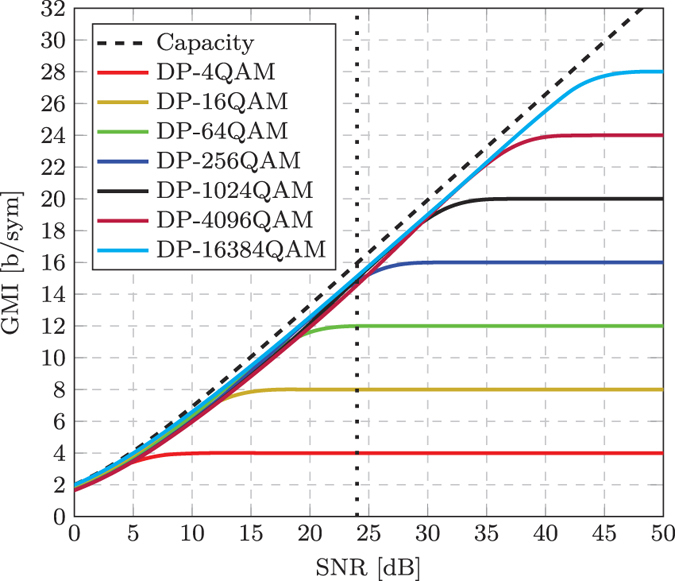
Simulation of GMI as a function of received SNR for the binary reflected Gray code labelled square QAM formats. The dashed line represents the Shannon capacity for an AWGN channel, while the dotted line represents the SNR limit (24 dB) of the state-of-the-art transceiver used in this work.

The multi-level drive signals required for each *m*QAM format were generated by combining 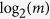 decorrelated random binary sequences of length 2^15^, where 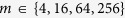. The coded bits were mapped to squared QAM constellations using a memoryless mapper based on the binary reflected Gray code, which generated the transmitted symbols, 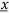, as shown at the output of the mapper in Fig. 1(b). The complex symbols were subsequently up-sampled to 4 Sa/sym, which provided a symbol rate of 8 GBd as the DAC operated at 32 GSa/s. The complex signals were filtered using a root raised cosine (RRC) filter with a roll-off factor of 1% and a stop-band attenuation in excess of 30 dB. The filtered *m*QAM signals were pre-emphasised to overcome the bandwidth response of the electrical and opto-electonic components in the transmitter, before being quantised to 64 discrete levels, which corresponded to the 6 physical bits of resolution available within the DAC. Finally, the I and Q components were loaded onto an FPGA and output using the DAC. For the super-channel transmission experiment, a second set of decorrelated I and Q components were loaded onto a second FPGA/DAC pair for modulation of the odd or even sub-carriers.

#### DSP at the Receiver

The received signals (sampled at 160 GS/s) were initially corrected for receiver skew imbalance and normalised to overcome the varying responsivities of the 70 GHz balanced photodiodes within the coherent receiver. For the super-channel experiment, each sub-carrier was downconverted to baseband before passing through the remaining DSP functions. For the single carrier experiment, the sub-carrier downconversion block was excluded from the DSP chain. Each polarisation was subsequently resampled to 2 Sa/sym before matched RRC filtering. A blind 51-tap radially directed equaliser (RDE)^24^ was used to equalise the signal and to undo polarisation rotations, with the constant modulus algorithm (CMA) equaliser^25^ used for tap-weight pre-convergence. The symbols at the output of the equaliser were decimated to 1 Sa/sym and the intermediate frequency was estimated and removed using a 4^th^-order nonlinearity algorithm^26^. The carrier phase was estimated per polarisation using a decision directed phase estimation algorithm and the complex field was averaged over a 64 T-spaced sliding window to improve the estimate^27^. Gram-Schmidt (GS) orthogonalisation^28^ was performed in order to correct for sub-optimal phase bias in the transmitter IQ modulators, which occurred over time due to temperature variations. The symbols at the output of the GS orthogonalisation stage represented the received symbols, 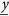, as shown in Fig. 1(b), and were used to calculate the SNR, MI, and BER_pre_, before being passed into the SD-FEC decoder described in^15^ to estimate the GMI, normalised GMI and BER_post_.

### SNR, MI and GMI Calculation

#### SNR

A memoryless AWGN channel was assumed in each polarisation, which assumes 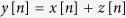, where 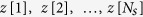 are independent and identically distributed zero-mean Gaussian random variables with (total) variance 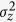. This variance was estimated based on the transmitted and received symbols (across both polarisations) as 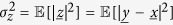. The received SNR 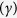 is given by 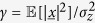, where 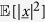 is the average received symbol energy.

#### MI

The MI per polarisation was calculated from the transmitted and received symbols using its definition:


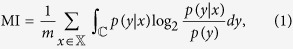


where 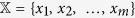 is the set of possible transmitted symbols. Due to the assumption of an AWGN channel, the channel law is given by:


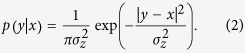


Using (2) in (1), the MI is approximated using the following Monte-Carlo estimate:


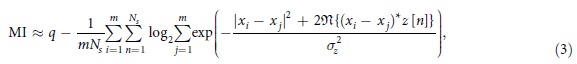


where 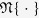 denotes the real part and 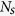 is the number of transmitted symbols.

#### GMI

In order to calculate the GMI, LLRs were initially calculated for each received complex symbol (at each discrete time index *n*) per polarisation via:


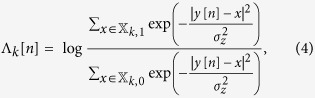


where 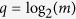 is the number of bits per constellation symbol, 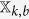 is the set of constellation symbols labelled with a bit 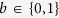, at bit position 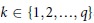. The GMI was approximated from the coded bits 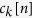 and the LLRs using Monte Carlo integration via:


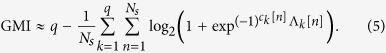


## Additional Information

**How to cite this article**: Maher, R. *et al.* Increasing the information rates of optical communications via coded modulation: a study of transceiver performance. *Sci. Rep.* **6**, 21278; doi: 10.1038/srep21278 (2016).
